# Does von Willebrand factor improve the predictive ability of current risk stratification scores in patients with atrial fibrillation?

**DOI:** 10.1038/srep41565

**Published:** 2017-01-30

**Authors:** Amaya García-Fernández, Vanessa Roldán, José Miguel Rivera-Caravaca, Diana Hernández-Romero, Mariano Valdés, Vicente Vicente, Gregory Y. H. Lip, Francisco Marín

**Affiliations:** 1 Cardiology Service, Alicante University General Hospital, Alicante Institute for Health and Biomedical Research (ISABIAL - FISABIO Foundation), Alicante, Spain; 2 Department of Hematology and Clinical Oncology, Morales Meseguer University Hospital, University of Murcia, Biohealth Research Institute Virgen de la Arrixaca, IMIB-Arrixaca, Murcia, Spain; 3 Department of Cardiology, Virgen de la Arrixaca University Hospital, University of Murcia, Biohealth Research Institute Virgen de la Arrixaca, IMIB-Arrixaca, Murcia, Spain; 4 University of Birmingham Institute of Cardiovascular Sciences, City Hospital, University of Birmingham, Birmingham, United Kingdom; 5 Aalborg Thrombosis Research Unit, Department of Clinical Medicine, Aalborg University, Aalborg, Denmark

## Abstract

Von Willebrand factor (vWF) is a biomarker of endothelial dysfunction. We investigated its role on prognosis in anticoagulated atrial fibrillation (AF) patients and determined whether its addition to clinical risk stratification schemes improved event-risk prediction. Consecutive outpatients with non-valvular AF were recruited and rates of thrombotic/cardiovascular events, major bleeding and mortality were recorded. The effect of vWF on prognosis was calculated using a Cox regression model. Improvements in predictive accuracy over current scores were determined by calculating the integrated discrimination improvement (IDI), net reclassification improvement (NRI), comparison of receiver-operator characteristic (ROC) curves and Decision Curve Analysis (DCA). 1215 patients (49% males, age 76 (71–81) years) were included. Follow-up was almost 7 years. Significant associations were found between vWF and cardiovascular events, stroke, mortality and bleeding. Based on IDI and NRI, addition of vWF to CHA_2_DS_2_-VASc statistically improved its predictive value, but c-indexes were not significantly different. For major bleeding, the addition of vWF to HAS-BLED improved the c-index but not IDI or NRI. DCA showed minimal net benefit. vWF acts as a simple prognostic biomarker in AF and, whilst its addition to current scores statistically improves prediction for some endpoints, absolute changes and impact on clinical decision-making are marginal.

Atrial fibrillation (AF) is increasingly more common and confers a five-fold increase in the risk of stroke^1^. AF patients also show high incidence of other cardiovascular events, such as acute coronary syndrome and cardiovascular death^2^ and a residual risk remains despite the use of oral anticoagulation (OAC)^3^. When compared to control, OAC with Vitamin K antagonists (VKA) reduces the risk of stroke by 64% and all-cause mortality by 26%, with further reductions using non-VKA OACs (NOACs)^4^,^5^.

The risks of thromboembolism and bleeding in AF are heterogeneous and several risk stratification schemes have been developed to tailor the decision-making. Guidelines^6^,^7^ recommend the use of the CHA_2_DS_2_-VASc score [Congestive heart failure, Hypertension, Age ≥ 75 years, Diabetes mellitus, Stroke, Vascular disease, Age 65 to 64 years, Sex category], as a simple, clinical risk factor-based approach to thromboprophylaxis^8^. This score has been validated in different cohorts and we have previously demonstrated that it is also predictive for vascular events and mortality in AF^9^. Nevertheless, the predictive value of the CHA_2_DS_2_-VASc and other clinical factor-based risk stratification schemes for identifying ‘high risk’ patients that develop events remains modest^10^. Nonetheless, the CHA_2_DS_2_-VASc has shown to reliably identify AF patients at truly low-risk of thromboembolism, who require no antithrombotic therapy^11^. However, use of CHA_2_DS_2_-VASc results in a high proportion of patients treated with OAC, and most would experience no events^12^. CHA_2_DS_2_-VASc also does not incorporate all possible risk factors for embolism, such as renal impairment, nor detailed echocardiographic or biochemical parameters^13^.

On the other hand, OAC increases the risk of bleeding complications, the most serious of which is intracranial haemorrhage^4^. Many stroke risk factors have also been identified as risk factors for bleeding^10^,^14^. Several risk stratification scores have been developed to estimate bleeding risk in AF^15^. For example, the HAS-BLED score (Hypertension, Abnormal renal/liver function, previous Stroke, previous Bleeding/predisposition, Labile INR, Elderly (age ≥65), concomitant Drugs or alcohol abuse), to assess the risk of major bleeding, and draw attention to revisable bleeding risk factors^16^.

Continuous efforts have been made to improve stroke and bleeding risk stratification in AF and various studies point out to a promising role of cardiac biomarkers to further refine these risks^17^,^18^.

Plasma glycoprotein von Willebrand factor (vWF) is synthesized mainly by endothelial cells in response to endothelial activation or damage, promoting platelet adhesion and aggregation at sites of vascular injury^19^. vWF has been considered an established marker of endothelial damage/dysfunction^20^. Increased plasma levels of vWF have been found in different inflammatory and atherosclerotic vascular diseases^21^, as well as in AF^22^,^23^, and is predictive of stroke and vascular events^24^. We have previously shown that high plasma vWF predicts adverse cardiovascular events, mortality and major bleeding in anticoagulated AF patients^25^.

In this study, we investigated the role of vWF on prognosis in relation to cardiovascular events, stroke and cardiovascular mortality, as well as major bleeding, in a large prospective ‘real-world’ cohort of anticoagulated patients with AF, and determined whether the addition of vWF to current clinical risk stratification schemes improved event-risk prediction.

## Methods

### Study patients

During the second semester of 2007 we recruited consecutive patients with non-valvular AF from our outpatient anticoagulation clinic in a tertiary hospital of south-eastern Spain. All the patients received VKA and needed to be stabilized for at least 6 months (international normalized ratio: 2.0–3.0), so at baseline the average time in therapeutic range (TTR) was 100%, to enable ‘anticoagulation status’ homogeneity of the baseline cohort. We excluded patients with valvular AF or prosthetic heart valves, as well as those with any acute coronary syndrome, stroke, hemodynamic instability, hospital admissions or surgical interventions in the preceding 6 months.

Data on baseline clinical characteristics were recorded at study entry. The CHA_2_DS_2_-VASc and the HAS-BLED scores were calculated using established definitions of the different risk factors, as previously described^8^,^26^. Follow-up was conducted by visits to our anticoagulation clinic. Adverse cardiovascular events were defined as follows: stroke/transient ischaemic attack, systemic and peripheral embolism, acute coronary syndrome, acute heart failure and cardiac death. The composite cardiovascular endpoint included all these events. Major bleeding events were assessed following the International Society of Thrombosis and Haemostasis criteria^27^. All-cause deaths were also recorded.

### Blood samples and laboratory analysis

At study inclusion, blood samples were drawn atraumatically and without stasis into syringes pre-loaded with trisodium citrate (0.011 mol/l). Platelet-poor plasma fractions were obtained by centrifugation at 4 °C for 20 min at 2,200 g. Aliquots were stored at −80 °C to allow batch analysis. Plasma vWF antigen levels were assessed in an automated coagulometer ACL Top 3G, HemosIL von Willebrand factor (Instrumentation Laboratory, Lexington, Massachusetts). The inter-assay and intra-assay coefficients of variation were 1.4% and 9%, respectively, and the lower limits of detection were 2.2 IU/dl and 21 ng/ml, respectively.

### Statistical analysis

Continuous variables were tested for normal distribution by the Kolmogorov-Smirnov test. Continuous variables are presented as a mean ± standard deviation or median (interquartile range), as appropriate, and categorical variables, as percentages. Receiver-operator characteristic (ROC) curve analyses (ie. c-indexes) were generated to test the predictive discrimination of vWF to identify association with adverse events during follow-up. The cut point with the best sensitivity and specificity for each adverse event was chosen. The independent effect of variables (vWF, CHA_2_DS_2_-VASc and HAS-BLED scores) on prognosis was calculated using a Cox proportional hazards regression model. Differences in event-free survival between patients with different levels of vWF were reflected by Kaplan-Meier curves.

To compare the predictive ability of risk-stratification schemes before and after adding vWF levels to the models, we calculated the statistical significance of the difference between the areas under the two ROC curves (AUC) with the method of DeLong *et al*.^28^. Also, improvement in the predictive accuracy of the models was evaluated by calculating the relative integrated discrimination improvement (IDI) and the net reclassification improvement (NRI), as described by Pencina *et al*.^29^.

We also estimated the clinical usefulness and net benefit of the new predictive models using decision curve analysis (DCA), as described by Vickers *et al*.^30^. This analysis identifies patients who will have any of the adverse events evaluated, based on the predictions of the modified risk score in comparison with the original. The x-axis shows threshold values for adverse event risk while the y-axis represents the net benefit for the different threshold values of adverse event risk. A higher net benefit is provided by those prediction models that are farthest away from the slanted dash grey line (i.e., assume all adverse events) and the horizontal black line (i.e., assume no adverse event).

A p value < 0.05 was accepted as statistically significant. Statistical analyses were performed using SPSS v. 15.0 (SPSS, Inc., Chicago, IL, USA), MedCalc v. 16.4.3 (MedCalc Software bvba, Ostend, Belgium) and STATA v. 12.0 (Stata Corp., College Station, TX, USA) for Windows.

### Ethical issues

This study was approved by the Ethical Committee of Morales Meseguer University Hospital and was performed in accordance with the ethical standards laid down in the 1964 Declaration of Helsinki and its later amendments. Patients gave their informed consent prior to their inclusion in the study.

## Results

We included 1215 patients with non-valvular AF. Baseline characteristics of the patients are shown in Table 1. During a median follow-up of 2373 (IQR 1564–2892) days, 115 patients presented stroke (1.45%/year); 498 patients died (6.30%/year) and 222 experienced major bleeding (2.81%/year). Annual rates of the different adverse events are summarized in Table 2.

### Univariate and multivariate analysis

For each adverse event, we constructed a ROC curve for vWF levels, that gave a median cut-off point of 190 UI/dL [AUC: 0.60 (95%CI: 0.56–0.64); p < 0.001] for the composite cardiovascular end-point; 194 UI/dL [AUC: 0.60 (95%CI: 0.55–0.65); p < 0.001] for stroke; 184 UI/dL [AUC: 0.62 (95%CI: 0.59–0.65); p < 0.001] for total mortality; 184 UI/dL [AUC: 0.64 (95%CI: 0.57–0.71); p < 0.001] for cardiovascular mortality and 197 UI/dL [AUC: 0.61 (95%CI: 0.57–0.65); p < 0.001] for major bleeding.

**Figure 1:**
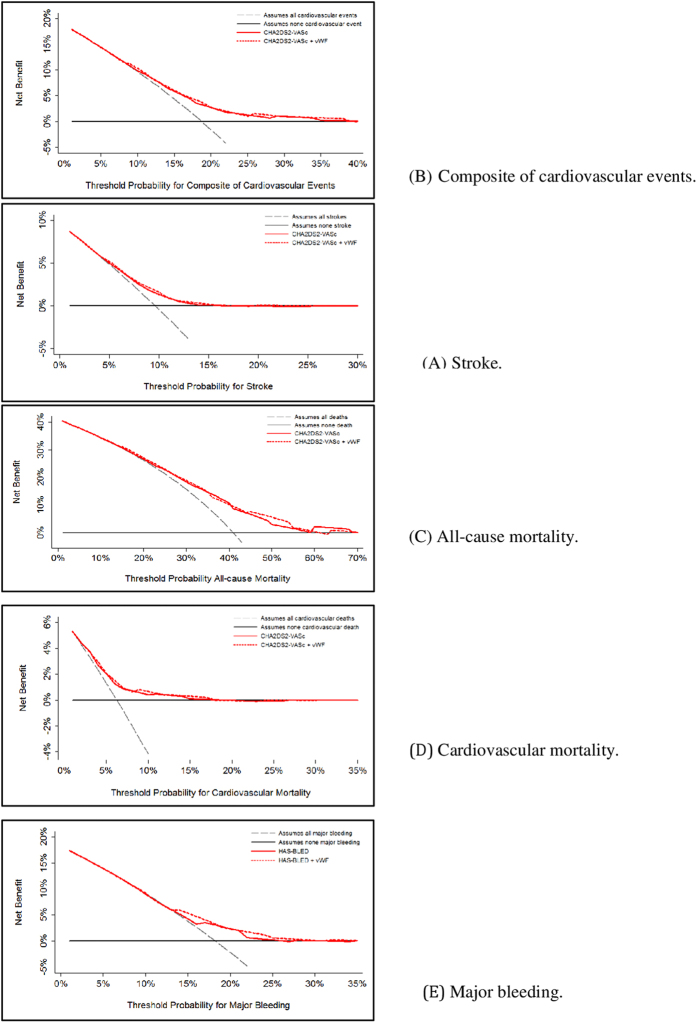
Decision curves for the original and modified risk scores (adding von Willebrand factor).

Univariate and multivariate analysis for the different adverse events for vWF levels and stroke and bleeding risk-stratification scores are shown in Table 3. On univariate analyses, plasma vWF levels were significantly predictive of cardiovascular events, stroke, all cause-mortality, cardiovascular death and major haemorrhage. CHA_2_DS_2_-VASc score significantly predicted the composite cardiovascular end-point, stroke, all-cause and cardiovascular death, whilst HAS-BLED score was an independent predictor of major bleeding. After adjustment for the CHA_2_DS_2_-VAScscore, vWF levels were significantly associated with the incidence of the composite cardiovascular end-point, stroke, all-cause mortality and cardiovascular mortality. Also, when we adjusted for HAS-BLED score, vWF was a significant predictor of major bleeding (Table 3). Figure 1 illustrates Kaplan-Meier plots for each outcome in relation to vWF levels (please see online supplement).

### Additive effect of von Willebrand factor levels to clinical risk scores

Based on IDI and NRI, the addition of vWF to CHA_2_DS_2_-VASc statistically improved its predictive value for cardiovascular events, stroke and cardiovascular mortality, but C-indexes were not significantly different and remained modest (approx. 0.6). Whilst statistically significant, the change in IDI and NRI was small (<1% increase) in its predictive ability for cardiovascular events, stroke and cardiovascular mortality (Table 4). For major bleeding, addition of vWF to HAS-BLED improved the c-index but not the IDI or NRI (Table 5).

DCA graphically shows minimal net benefit of the modified CHA_2_DS_2_-VASc and HAS-BLED scores, that does not justify their clinical use or impact on practical decision-making (Fig. 1).

## Discussion

In this study of a large contemporary ‘real world’ cohort of stable anticoagulated non-valvular AF patients, we show that even if vWF acts as a simple prognostic biomarker and that the addition of vWF levels to the CHA_2_DS_2_-VASc and the HAS-BLED scores statistically improved prediction for some endpoints, absolute changes and clinical improvement or impact on clinical decision-making (using DCA) was marginal, with c-indexes all remaining modest (approx. 0.6).

Cardiac biomarkers have evolved as prognostic tools in different cardiovascular scenarios. The importance of vWF to cardiovascular disorders has been long recognised^31^. Raised levels of vWF have been described in AF individuals, compared to those in sinus rhythm^23^. Also, the prognostic role of plasma vWF in AF has been well-established^32^,^33^. In the Rotterdam Study, which included more than 6000 participants, the risk of stroke was associated with vWF levels in the general population, no matter AF was present or not^34^. Also, vWF appears to be able to identify, not only patients with high thromboembolic risk, but also subjects at risk of major bleeding and mortality. Indeed, major bleedings and death occur more frequently than embolism in anticoagulated AF patients, and in this study we show that bleeding rates doubled rates of stroke, and mortality was four times greater.

Other biomarkers, such us Growth Differentiation factor-15 (GDF-15, a marker of oxidative stress and inflammation) and high-sensitivity Troponin T (hs-TnT), have also been shown to act as additive prognostic markers (at least statistically) for major bleeding and death in AF subjects with OAC^35^,^36^.

In the present study, we demonstrate that raised vWF levels doubled the risk of stroke, cardiovascular death and major bleeding, and increased (by more than 50%) cardiovascular events and all-cause mortality. We have previously shown that this biomarker acts as an independent risk factor for adverse events, including cardiovascular events (stroke, acute coronary syndrome and acute heart failure), cardiovascular death, all cause mortality and major haemorrhagic episodes in a ‘real-life’ anticoagulated non-valvular AF population^25^. In another study, raised vWF activation factor levels (a modified test that detects vWF with high platelet affinity) correlated with all-cause mortality and cardiovascular events, but no association was observed with bleeding complications^37^. The same group had previously described an increased risk of bleeding complications, cardiovascular and all-cause mortality in patients with high levels of vWF during treatment with warfarin; nonetheless, only one third of the subjects in both studies had AF as the indication for OAC^38^.

Currently, the CHA_2_DS_2_-VASc score is recommended to assist clinical decision-making for OAC^6^,^7^, but its predictive value is modest^8^. In order to improve stroke risk stratification, composite risk prediction scores (CHA_2_DS_2_-VASc + HAS-BLED or CHADS_2_ + HAS-BLED) have been compared with separate individual scores, but they have not shown to significantly improve clinical prediction of thromboembolism and bleeding^39^. Unsurprisingly, cardiac biomarkers could help to refine event-risk stratification in these patients. For example, Lip *et al*. first showed that plasma vWF levels added to the CHADS_2_ and the Birmingham (precursor of CHA_2_DS_2_-VASc) schemes could help to refine risk stratification for vascular events (ischemic stroke, myocardial infarction or vascular death) in AF patients enrolled in the SPAF III trial^17^; however, they were not able to identify an independent association with the risk of stroke. Recently, Hijazi *et al*. demonstrated that N-terminal fragment B-type Natriuretic Peptide (NT-proBNP) and hs-TnT improved risk stratification for stroke, cardiac death and bleeding beyond the CHA_2_DS_2_-VASc score^36^,^40^. This group then proposed the ABC score (which adds biomarkers to clinical factors) in a selected clinical trial cohort of AF patients anticoagulated with apixaban or warfarin from the ARISTOTLE (Apixaban for Reduction in Stroke and Other Thromboembolic Events in Atrial Fibrillation)^41^. In a similar manner, the ABC-bleeding score was also validated, including biomarkers (haemoglobin, hsTnT and GDF-15 or cystatin C/glomerular filtration rate) and this score performed better than the (clinical factor-based) HAS-BLED or ORBIT scores^18^. Whilst statistically significant improvement in prediction was seen, overall c-indexes still remain modest (approx. 0.6–0.7).

Contrary to this evidence from the highly selected trial patient cohorts, we have not been able to demonstrate that the addition of a biomarker (specifically, vWF) to clinical risk scores markedly improves decision making for event risk prediction, as DCA only showed minimal net benefit of the modifiedscores that incorporated biomarkers. Nonetheless, our study adds some novel strengths to the current knowledge in this field, amongst ‘real-world’ AF patients. In light of our data, even if vWF levels provide independent prognostic information, we cannot recommend the addition of vWF as a biomarker to the CHA_2_DS_2_-VASc and HAS-BLED risk scores in order to refine event-risk prediction.

More insights are needed to explore the role of cardiac biomarkers to aid clinical decision-making for thromboprophylaxis in AF. The continued preoccupation with trying to improve prediction of ‘high risk’ patients with ever more complex scores (and often multiple biomarkers), with only marginal improvement in predictive performance, at the cost of simplicity and practicability, would seem counterintuitive for everyday clinical management^42^. Meanwhile, a more simple strategy, using conventional clinical risk scores, should be the preferred option. In short, simplicity and practicability (and costs of biomarkers) should be balanced.

## Limitations

Only a single determination of plasma vWF levels was made, at study entry, thus we cannot be sure if making other determinations during follow-up could have changed our results. Only patients on VKA were included, so our results may not be generalized to individuals receiving NOACs. TTR was 100% at study entry, but we do not have data of this parameter over the follow-up period, which could affect the occurrence of the adverse events. Although vWF levels are known to be associated to cardiovascular risk factors (such as hypertension or heart failure), we have previously shown that the association of this biomarker with adverse events in AF is independent of other clinical variables^25^.

## Conclusions

In conclusion, vWF acts as a simple prognostic biomarker in anticoagulated AF patients and, whilst addition of vWF levels to the CHA_2_DS_2_-VASc and the HAS-BLED scores statistically improved prediction for some endpoints, the absolute changes and clinical value or impact on practical decision-making was minimal.

## Additional Information

**How to cite this article**: García-Fernández, A. *et al*. Does von Willebrand factor improve the predictive ability of current risk stratification scores in patients with atrial fibrillation? *Sci. Rep.* **7**, 41565; doi: 10.1038/srep41565 (2017).

**Publisher's note:** Springer Nature remains neutral with regard to jurisdictional claims in published maps and institutional affiliations.

## Supplementary Material

Supplemental Information

## Figures and Tables

**Table 1:** Baseline characteristics of atrial fibrillation patients (N = 1215).

	Median (IQR) N (%)
Age	76 (71–81)
Male sex	599 (49.3)
Hypertension	1002 (82.5)
Diabetes	320 (26.4)
Heart failure	378 (31.1)
Ischaemic heart disease	231 (19.0)
Smoking	183 (15.1)
Dyslipemia	393 (32.3)
Previous stroke	224 (18.4)
Previous bleeding	102 (8.4)
Alcohol abuse	39 (3.2)
Renal disease	125 (10.3)
Hepatic disease	14 (1.2)
Antiplatelet drugs	216 (17.8)
CHA_2_DS_2_-VASc score	4 (3–5)
HAS-BLED score	2 (2–3)
vWF levels (UI/dL)	189.7 (132.0–234.0)

IQR: *interquartile range.*

**Table 2:** Annual rate of the different adverse events.

	n (%)	Annual rate (%)
Composite cardiovascular end-point^*^	226 (18.6)	2.90
Stroke	115 (9.5)	1.45
Total mortality	498 (41.0)	6.30
Cardiovascular death	76 (6.3)	0.96
Major bleeding	222 (18.3)	2.81
Intracranial haemorrhage	65 (5.4)	0.82
Mortality of bleeding episodes	45 (3.7)	0.56

^*^Stroke-thromboembolism, acute coronary syndrome, acute heart failure and cardiovascular death.

**Table 3:** Cox analysis for vWF levels, the CHA_2_DS_2_-VASc and the HAS-BLED scores for the different endpoints.

		Univariate analysis HR (95% CI)	Multivariate analysis HR (95% CI)
**Composite cardiovascular end-point**^**1**^	CHA_2_DS_2_-VASc score	1.36 (1.26–1.48); p < 0.001	1.34 (1.23–1.46); p < 0.001
	vWF > 190 UI/dL	1.96 (1.50–2.57); p < 0.001	1.77 (1.35–2.32); p < 0.001
**Stroke**^**1**^	CHA_2_DS_2_-VASc score	1.35 (1.20–1.51); p < 0.001	1.32 (1.18–1.49); p < 0.001
	vWF > 194 UI/dL	2.19 (1.50–3.20); p < 0.001	1.98 (1.36–2.90); p < 0.001
**All-cause mortality**^**1**^	CHA_2_DS_2_-VASc score	1.33 (1.26–1.40); p < 0.001	1.30 (1.23–1.38); p < 0.001
	vWF > 184 UI/dL	1.75 (1.46–2.10); p < 0.001	1.53 (1.28–1.84); p < 0.001
**Cardiovascular death**^**1**^	CHA_2_DS_2_-VASc score	1.49 (1.30–1.71); p < 0.001	1.46 (1.27–1.68); p < 0.001
	vWF > 184 UI/dL	2.34 (1.44–3.80); p < 0.001	1.95 (1.20–3.18); p = 0.007
**Major bleeding**^**2**^	HAS-BLED score	1.45 (1.28–1.65); p < 0.001	1.39 (1.23–1.58); p < 0.001
	vWF > 197 UI/dL	2.13 (1.62–2.79); p < 0.001	1.95 (1.49–2.57); p < 0.001

HR: *hazard ratio.*

^1^Variables included in multivariate analysis: CHA_2_DS_2_-VASc score and vWF plasma levels.

^2^Variables included in multivariate analysis: HAS-BLED score and vWF plasma levels.

**Table 4:** Comparison of the ROC curve, IDI and NRI of the modified CHA_2_DS_2_-VASc *vs*. original CHA_2_DS_2_-VASc in predicting endpoints.

	C-index	95% CI *p*	p*	IDI	*p*	NRI	*p*
(A) Composite cardiovascular end-point							
CHA_2_DS_2_-VASc	0.630	0.603–0.658 < 0.001	0.111	0.0065	<0.001	−0.016	0.012
CHA_2_DS_2_-VASc+vWF	0.641	0.613–0.668 < 0.001					
(B) Stroke							
CHA_2_DS_2_-VASc	0.610	0.582–0.637 p < 0.001	0.131	0.0037	0.040	−0.017	<0.001
CHA_2_DS_2_-VASc+vWF	0.623	0.595–0.650 p < 0.001					
(C) All-cause mortality							
CHA_2_DS_2_-VASc	0.661	0.634–0.688 p < 0.001	0.076	0.0091	0.048	0.001	0.864
CHA_2_DS_2_-VASc+vWF	0.670	0.643–0.697 p < 0.001					
(D) Cardiovascular mortality							
CHA_2_DS_2_-VASc	0.656	0.628–0.683 p < 0.001	0.156	0.0041	0.033	−0.018	0.004
CHA_2_DS_2_-VASc+vWF	0.670	0.643–0.697 p < 0.001					

CI = confidence interval; IDI = integrated discriminatory improvement; NRI = net reclassification improvement.

^*^For c-index comparison.

**Table 5:** Comparison of the ROC curve, IDI and NRI of the modified HAS-BLED *vs*. original HAS-BLED in predicting major bleeding.

	C-index	95% CI *p*	p*	IDI	*p*	NRI	*p*
HAS-BLED	0.592	0.564–0.620 p < 0.001	0.025	0.0105	0.056	0.012	0.735
HAS-BLED+vWF	0.614	0.586–0.641 p < 0.001					

CI = confidence interval; IDI = integrated discriminatory improvement; NRI = net reclassification improvement.

^*^For c-index comparison.
