# Supplementary material for: Does von Willebrand factor improve the predictive ability of current risk stratification scores in patients with atrial fibrillation?
Source: Sci Rep. 2017 Jan 30;7:41565. doi: 10.1038/srep41565 (PMC5278507; doi:10.1038/srep41565)
Supplement: Supplemental Information [file srep41565-s1.pdf]

Does von Willebrand factor improve the predictive ability of current risk stratification scores in patients with atrial fibrillation?

Amaya García-Fernández[1], MD, PhD; Vanessa Roldán[2], MD, PhD; José Miguel Rivera-Caravaca[3], MSc, RN; Diana Hernández-Romero[3], PhD; Mariano Valdés[3], MD, PhD; Vicente Vicente[2], MD, PhD; Gregory Y H Lip*[4], MD; Francisco Marín*[3], MD, PhD.

[*joint senior authors]

[1]Cardiology Service, Alicante University General Hospital, Alicante Institute for Health and Biomedical Research (ISABIAL - FISABIO Foundation), Alicante, Spain.

[2]Department of Hematology and Clinical Oncology. Morales Meseguer University Hospital. University of Murcia. Biohealth Research Institute Virgen de la Arrixaca, IMIB-Arrixaca. Murcia, Spain.

[3]Department of Cardiology. Virgen de la Arrixaca University Hospital. University of Murcia. Biohealth Research Institute Virgen de la Arrixaca, IMIB-Arrixaca. Murcia, Spain.

[4]University of Birmingham Institute of Cardiovascular Sciences. City Hospital, University of Birmingham, Birmingham, United Kingdom, and Aalborg Thrombosis Research Unit, Department of Clinical Medicine, Aalborg University, Aalborg, Denmark.

Corresponding author

Vanessa Roldán

Department of Hematology and Clinical Oncology

Hospital Universitario Morales Meseguer

Av Marqués de los Vélez, s/n, 30008 Murcia

Tel: 0034 968 36 09 00 e-mail: vroldans@gmail.com

ONLINE SUPPLEMENT

Supplemental Figure.

Figure I. Kaplan-Meier analysis for each adverse event in relation to vWF levels.

(A) Composite of cardiovascular events[1].

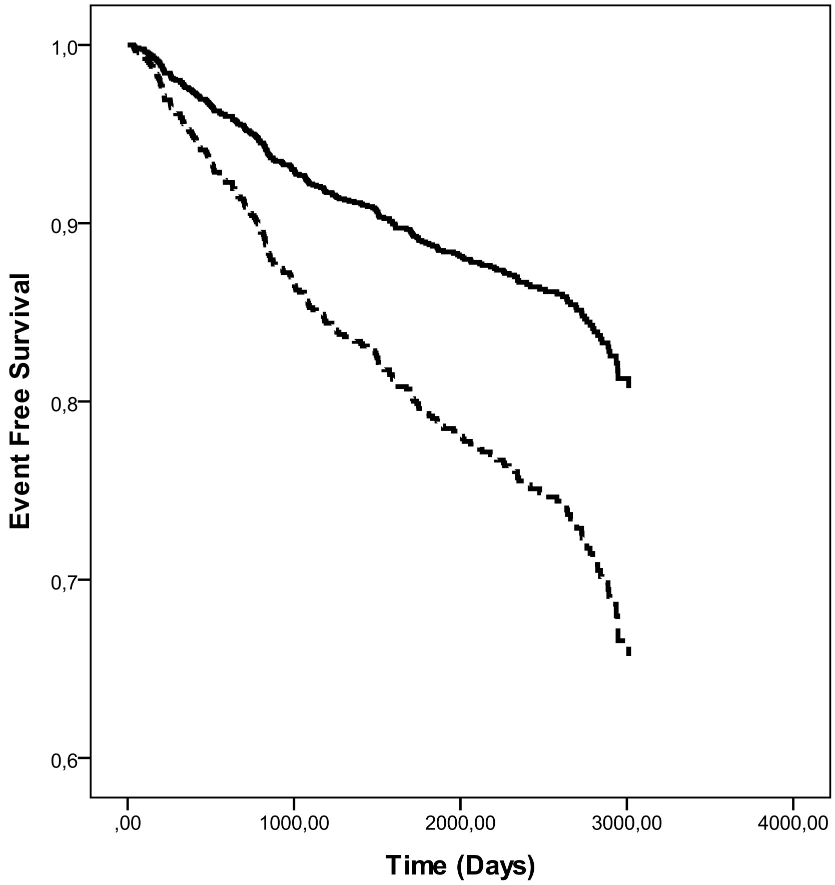

p<0.001

[1]Solid line indicates vWF <190UI/dL; Dash line indicates vWF ≥190 UI/dL.

(B) Stroke[1].

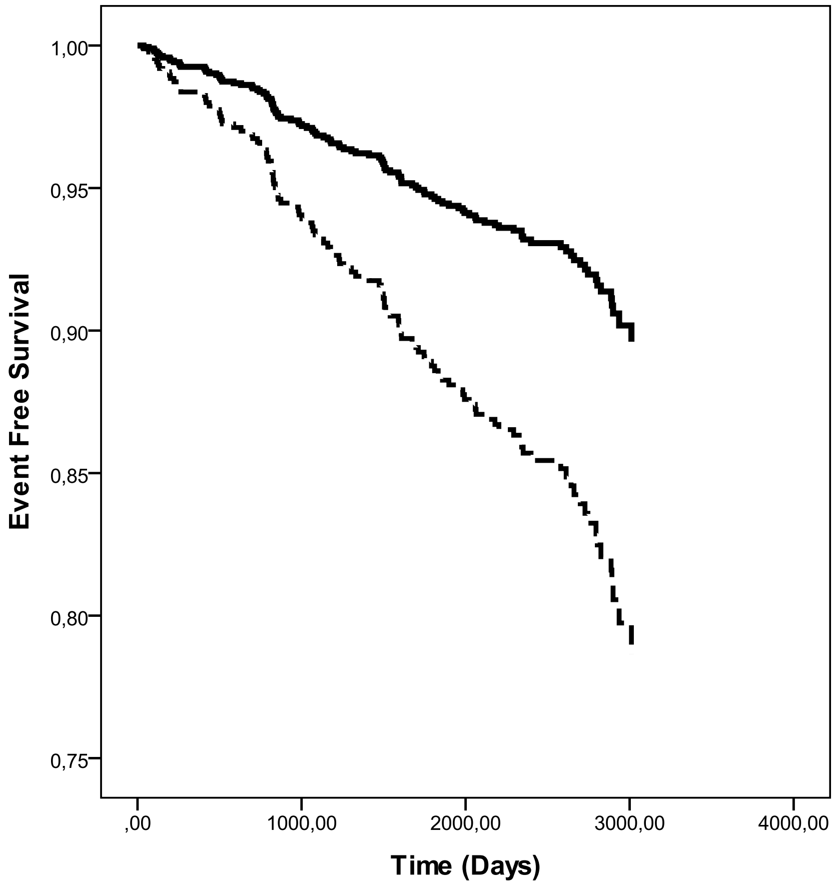

p<0.001

[1]Solid line indicates vWF <194UI/dL; Dash line indicates vWF ≥194 UI/dL.

(C) Total mortality[1].

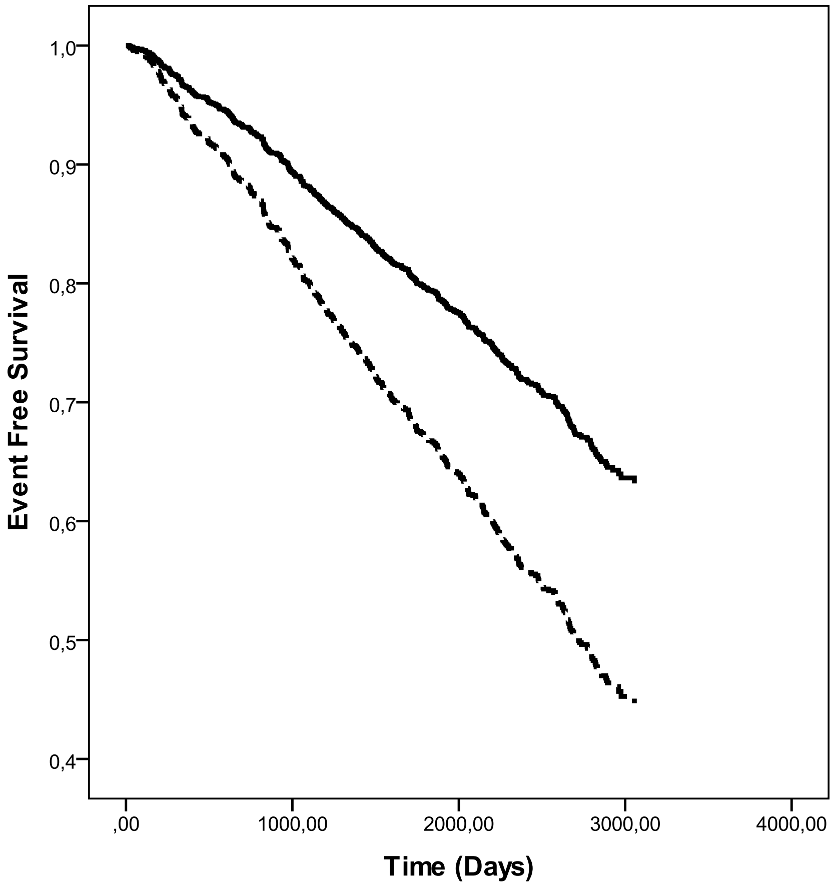

p<0.001

[1]Solid line indicates vWF <184UI/dL; Dash line indicates vWF ≥184 UI/dL.

(D) Cardiovascular mortality[1].

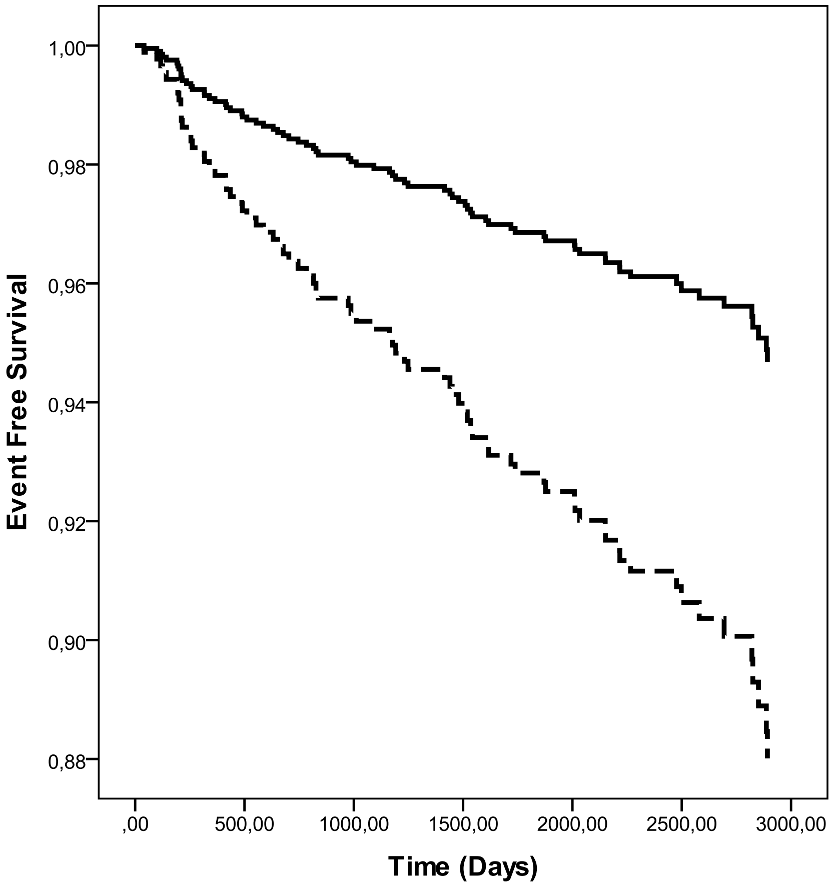

p<0.001

[1]Solid line indicates vWF <184UI/dL; Dash line indicates vWF ≥184 UI/dL.

(E) Major bleeding[1].

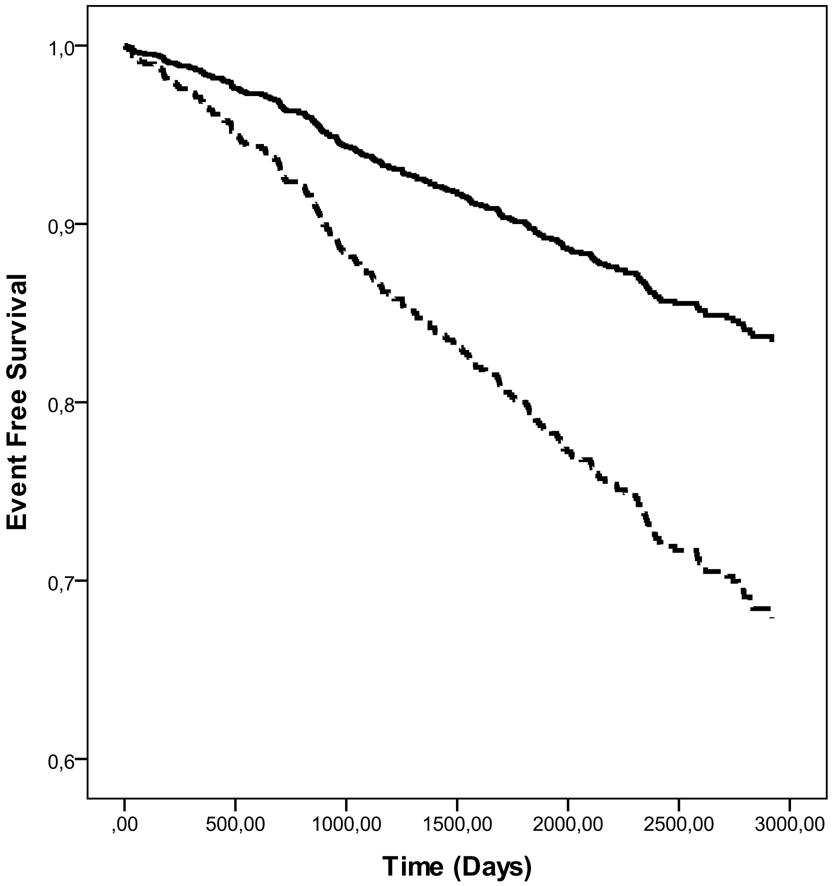

$p<0.001$

[1]Solid line indicates vWF <197UI/dL; Dash line indicates vWF ≥197 UI/dL.
